# Ultrafine particles: unique physicochemical properties relevant to health and disease

**DOI:** 10.1038/s12276-020-0405-1

**Published:** 2020-03-17

**Authors:** Hyouk-Soo Kwon, Min Hyung Ryu, Christopher Carlsten

**Affiliations:** 1 Department of Allergy and Clinical Immunology, Asan Medical Center, University of Ulsan College of Medicine, Seoul, Republic of Korea; 2 Air Pollution Exposure Laboratory, Division of Respiratory Medicine, Faculty of Medicine, The University of British Columbia, Vancouver, BC Canada

**Keywords:** Medical research, Pathogenesis

## Abstract

Ultrafine particles (UFPs) are aerosols with an aerodynamic diameter of 0.1 µm (100 nm) or less. There is a growing concern in the public health community about the contribution of UFPs to human health. Despite their modest mass and size, they dominate in terms of the number of particles in the ambient air. A particular concern about UFPs is their ability to reach the most distal lung regions (alveoli) and circumvent primary airway defenses. Moreover, UFPs have a high surface area and a capacity to adsorb a substantial amount of toxic organic compounds. Harmful systemic health effects of PM_10_ or PM_2.5_ are often attributable to the UFP fraction. In this review, we examine the physicochemical characteristics of UFPs to enable a better understanding of the effects of these particles on human health. The characteristics of UFPs from diesel combustion will be discussed in the greatest detail because road vehicles are the primary source of UFP emissions in urban pollution hotspots. Finally, we will elaborate on the role of UFPs on global climate change, since the adverse effects of UFPs on meteorological processes and the hydrological cycle may even be more harmful to human health than their direct toxic effects.

## Air quality: tiny particles with big effects

Ultrafine particles (UFPs) from auto exhaust, factory emissions, and woodburning negatively affect human health and can alter weather patterns. UFPs, particles less than 100 nanometers, smaller than the smallest bacterium, are the most common airborne particles. Their size allows them to penetrate the deepest lung passageways, sometimes carrying toxic metals or organic compounds that trigger inflammation and disease. Hyouk-Soo Kwon at the University of Ulsan, Seoul, South Korea, and coworkers have reviewed the sources and effects of UFPs. Auto engines are a primary source; recent improvements in combustion technology have resulted in production of smaller particles, with worse effects on health. UFPs have also been found to affect cloud formation and behavior, altering rainfall patterns and potentially causing flooding or drought. Understanding the properties of UFPs will help find ways to mitigate their effects.

## Introduction

Ultrafine particles (UFPs) are particles with an aerodynamic diameter of 0.1 µm (100 nm) or less^1,2^. Particles may be described in terms of surface area per particle, in terms of particle number (PN) or mass, or in terms of the concentration of either of those metrics within an aerosol volume. The metrics that are most commonly used to describe particulate matter (PM) are the number concentration and the mass concentration. Coarse particulate matter, with an aerodynamic diameter of 10 μm or less (PM_10_), and fine particulate matter, with a diameter of 2.5 μm or less (PM_2.5_), are typically described in terms of mass distribution. In contrast, UFPs have negligible mass but are the dominant contributor to the total number of particles in the atmosphere and are thus better quantified by number concentration^3^.

**Fig. 1:**
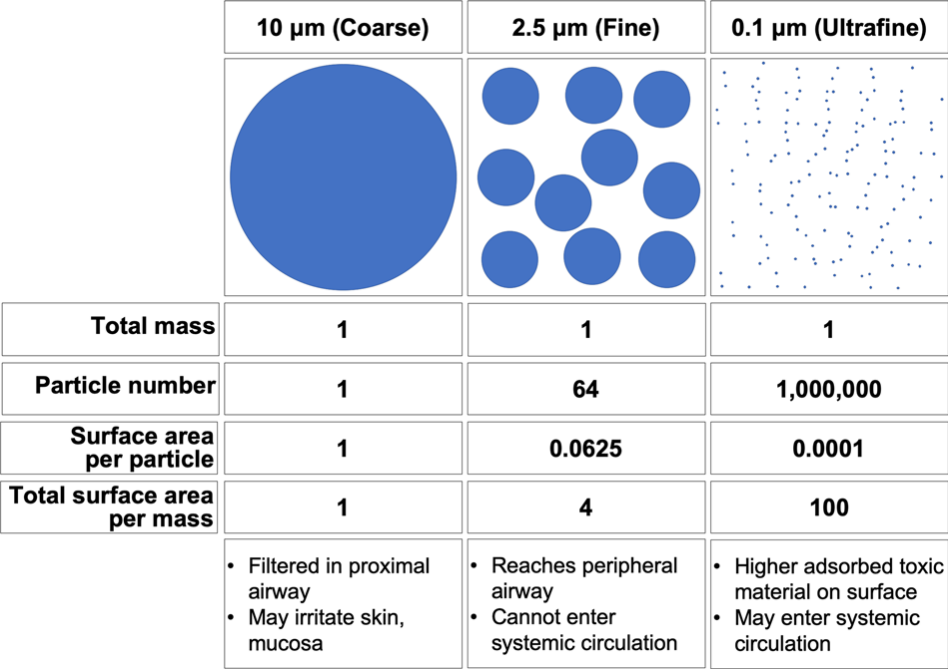
Comparison of the surface area of particles with different diameters. The diagram assumes that all particles in each category are perfect spheres, have the same density, and are present in an equal amount of mass. The mass, particle number, and surface area of coarse particles are all arbitrarily designated as 1. Other numbers are relative to the coarse particle. The large surface area and ability to enter circulation are the two most significant characteristics of ultrafine particles that make them more toxic than other larger particles.

A particular concern about UFPs is their ability to reach the most distal lung regions (alveoli) and circumvent primary airway defenses. When inhaled, UFPs can pass through the respiratory tract with high efficiency down to the alveoli due to their small size. A small fraction of UFPs penetrate the alveolar–capillary barrier and can thus be distributed throughout the body via the circulatory system^4^. Because of this property of UFPs, extrapulmonary diseases related to PM exposure may be particularly attributable to UFPs^1,5^. Furthermore, UFPs are thought to be more threatening than larger PM due to their higher specific surface area (total exposed surface area per unit of mass). Large surface area and high surface reactivity enable UFPs to adsorb, for a given mass of PM, greater quantities of hazardous metals, and organic compounds that can generate oxidative stress (Fig. 1)^5,6^.

A recent development in filtration technologies for reducing PM emissions has significantly improved the air quality in many large cities. However, UFPs can still be formed from the condensation of semivolatile organic compounds (SVOCs) in the gas phase that bypass the filtration technology. These additional UFPs from SVOCs, which are mostly toxic organic aerosols (OAs)^7^, may outnumber the filterable solid particles in emissions, especially in industrial stack emissions^8,9^. Consequently, despite the improvement in overall ambient air quality, exposure to toxic UFPs may have increased for those who spend a considerable amount of time near emission sources. Moreover, modern fossil fuel combustion technologies, primarily related to diesel, emit particles that can adsorb more reactive oxygen species with the functional groups on the particle surface, making the UFPs more toxic^10^.

Beyond the direct impact of UFPs on health, an important unanticipated implication of UFP emissions from fossil fuel combustion is their impact on precipitation. UFPs in the atmosphere do not necessarily change the total precipitation amount, but change the precipitation’s spatial and intensity distribution^11^. Increased emission of UFPs relative to fine particles can result in less steady rain or more extended drought periods in some areas, and heavy torrential rain and vigorous flooding in other areas thousands of kilometers away, impacting public health globally^11–13^.

## Aerosols in the atmosphere

**Fig. 2:**
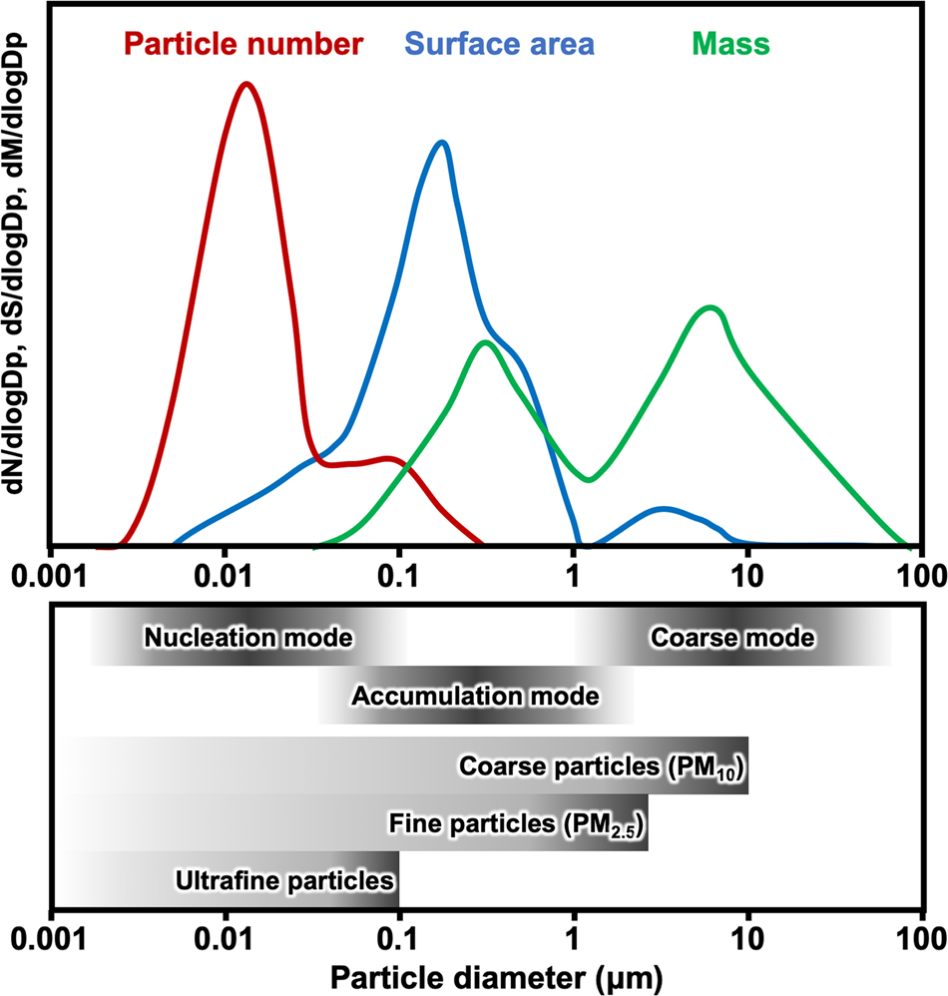
Particle-size distribution of atmospheric PM. A schematic representation of a typical ambient particle-size distribution of the number concentration, surface area concentration, and mass concentration (dN/dLog Dp, particle number per cubic millimeter; dS/dLog Dp, particle surface area per cubic millimeter; and dM/dLog Dp, particle mass per cubic millimeter, respectively)^99^. Vertical scaling is individual to each distribution. There are three modes of atmospheric aerosol particles: nucleation mode, accumulation mode, and coarse mode. Ultrafine particles (UFPs) are all particles with a diameter of 100nm or less. Most nucleation mode particles and the fraction of accumulation mode particles are UFPs.

**Fig. 3:**
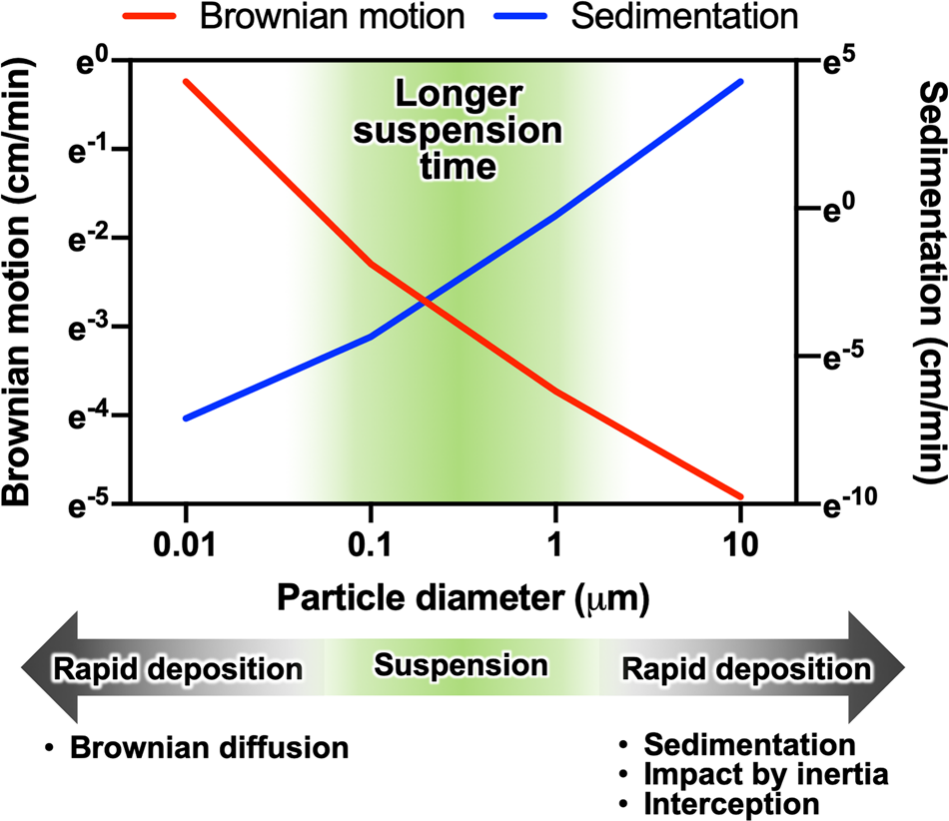
Deposition of particles according to size distribution. Ultrafine particles are rapidly deposited by Brownian diffusion, especially particles <20 nm. Large coarse particles are deposited by sedimentation, impact by inertia, and interception. Particles between 30 nm and 1 μm tend to have longer atmospheric lifetimes because they are less likely to be deposited in either way. These particles accumulate in the atmosphere due to the long suspension time and are thus called the accumulation mode.

Three modes in the particle-size distribution are the nucleation mode, accumulation mode, and coarse mode^14^ (Fig. 2). The nucleation mode corresponds to particles that have been formed from gaseous molecules, usually with sizes <50 nm, and have later grown via the condensation of other gaseous molecules and coagulation with other nucleated particles^15^. Nucleation of new particles is frequently observed in forested areas, where emissions of biogenic volatile organic compounds (VOCs) are high and existing suspended particles are low in number^16^. The higher number and larger surface area for a given mass concentration in this mode favor the occurrence of condensation and coagulation processes. The accumulation mode comprises particles between 0.1 μm and 1 μm, resulting from the emissions of fine particles and dynamic events, such as condensation and coagulation. The accumulation mode is so named because particle removal mechanisms are least efficient in this fraction, causing particles to accumulate and have a long lifetime, typically 7–30 days, until they are ultimately lost through rain or other forms of precipitation (Fig. 3). The coarse mode consists mostly of large particles emitted via mechanical processes^17^.

**Fig. 4:**
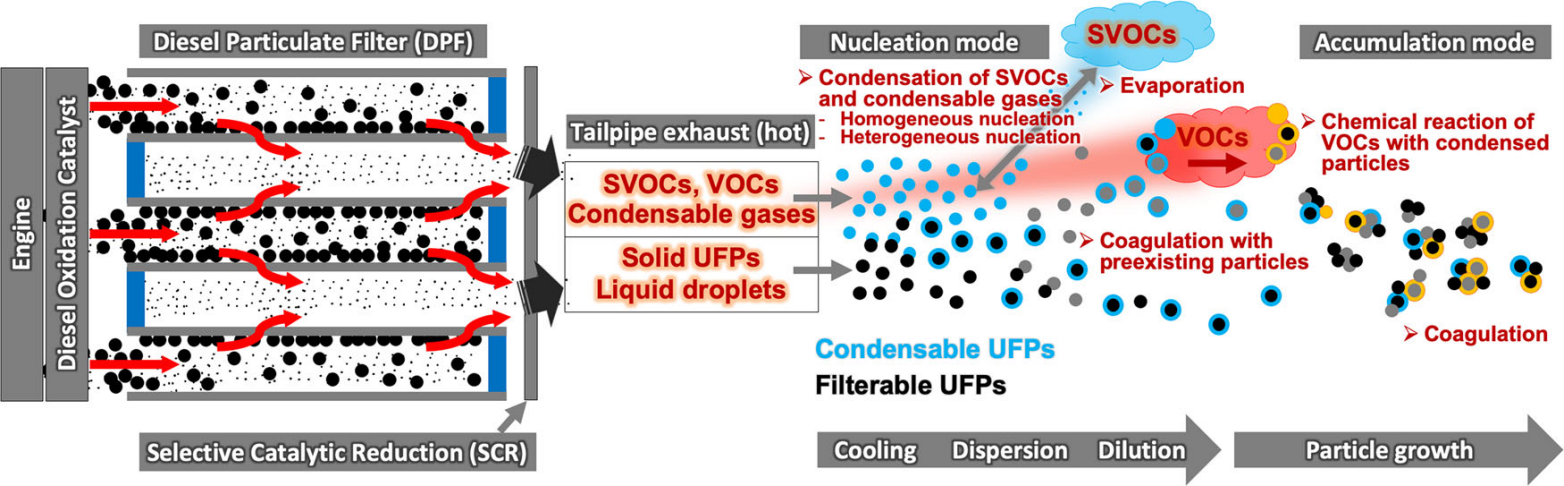
The dynamics of ultrafine particles emitted from a typical diesel engine. A significant amount of volatile organic compounds (VOCs) and semivolatile organic compounds (SVOCs) are catalyzed, and removed within the diesel oxidation catalyst (DOC). Most of the coarse and fine filterable particles are filtered within the diesel particulate filter (DPF). Solid ultrafine particles (UFPs), VOCs, SVOCs, and other condensable gases that escape the DOC and DPF are emitted via the tailpipe. As they are released, the hot gas cools, and SVOCs and some gases condense to form tiny nucleation mode particles. The process of vapors condensing homogeneously upon cooling to form UFPs, usually <50 nm, is called homogeneous nucleation. The process of vapors condensing on the surface of other existing UFPs or larger particles is called heterogeneous nucleation. When the exhaust gas is dispersed and diluted in ambient air, UFPs coagulate to form larger particles. Some condensed SVOCs evaporate and return to the gas phase; some coagulate with preexisting fine particles in the ambient air. The coagulation and evaporation process rapidly diminishes the particle number of the nucleation mode. VOCs that were emitted together may condense on the liquid aerosols and form secondary organic aerosols by chemical reactions. Particles grow in size, agglomerate, and thus increase the number of accumulation modes.

Condensation is the process of transferring gaseous molecules into nucleation mode particles or toward an existing particle. High condensation activity is observed in the exhaust tailpipe of diesel cars (Fig. 4). The condensation rate is highest for particles with diameters ranging from 0.03 to 0.3 μm, which corresponds mainly to the UFP fraction. The reverse process is evaporation, which is the transfer of molecules from the particle toward the gas phase. Changes in the concentration in the gas phase, temperature, pressure, or relative humidity affect the events of condensation and evaporation^2,18^.

Coagulation is the collision and formation of a single particle from two original particles. The UFPs experience Brownian motion, while larger fine particles provide large collision targets. Freshly nucleated UFPs coagulate readily with coexisting particles. Therefore, coagulation can be viewed as a main process for removing UFPs from the ambient air. As a result, coagulation is an important process for the deposition of UFPs but has little effect on PM mass concentrations^17,19^.

## Dynamics of UFPs

An essential feature of UFPs is their fast evolution, particularly of their smallest fractions <20 nm. These particles move under Brownian motion with movement based on diffusion via concentration gradients, so in situations with a high number concentration near emission sources, UFPs readily collide with adjacent particles to coagulate into larger particles or deposit on available surfaces (Fig. 3)^2^. Additionally, particle growth occurs primarily due to coagulation and condensation of SVOCs on the particle surface^20^. Consequently, UFPs have very short atmospheric lifetimes, usually approximately a few hours, and their concentrations quickly decay with increasing distances from the emission sources^21^. These features prompt three significant considerations when monitoring UFP exposures. The first is that individual exposures to UFPs should be assessed in the microenvironments, where individuals reside or pass through, rather than relying on measurements performed at fixed monitoring stations. The second is that the time resolution of particle measurement should be on the time scale of their evolution to capture the transient spike emissions from their sources^22^. The third is that condensable UFPs should be accounted for in the contribution to the total burden of UFPs, which are not often considered in certain emission sectors. Occupational groups who work in the vicinity of the source of diesel exhaust (DE)—such as professional drivers, firefighters, farmers, miners, railroad workers, heavy equipment operators, vehicle mechanics, tunnel workers, dockworkers, and shipping engineers—are exposed to high numbers of newly formed condensable and filterable UFPs (ref. ^23^). The number concentration of UFPs drops very sharply away from the source of emission due to both evaporation of the condensable UFPs and rapid coagulation of the particles^24^. Thus, the exposure to UFPs from DE may be significantly underestimated unless the number concentration measurement of the UFPs is made in high spatiotemporal resolution, in the vicinity of the source.

## Condensable UFPs

Total PM (TPM) emissions from sources include both filterable PM (FPM) and condensable PM (CPM). FPM is directly emitted from the source as solid or nonvolatile liquid particles and can be captured on a filter. CPM denotes particles that are initially in the vapor phase after combustion of the source, but immediately condense upon cooling in the ambient air and form solid salts or liquid droplets. It is important to note that CPM and OAs initially form as UFPs (ref. ^8^). Air quality monitoring stations, which mostly detect PM concentrations at ambient air temperature, measure both FPM and CPM. However, the measurement of PM concentrations in heated air, which is often the case within industrial stacks, will only include FPM. Thus, CPM emissions are not measured in particular source sectors, and the TPM can be underestimated. The underestimation of TPM explains in part why there is a gap between modeled and observed PM, especially during cold seasons^25^.

With the rapid development of technology to control FPM at the source, the FPM contribution to TPM is decreasing^26^, making CPM perhaps even more critical^27^. However, analysis methods for CPM are not as well developed as those for FPM, and even the more common Method 202 and OTM-37 have considerable shortcomings. Recent official emission inventories in the US (ref. ^28^) and Europe^29^ are meant to include both FPM and CPM emissions, but are very limited due to insufficient data since CPM is not measured in the emission surveys of many countries and is therefore not monitored routinely for most emission inventories^30–32^. Studies have shown that inorganic compounds, mostly sulfate, have been the dominant contributors to CPM (ref. ^9^), although recent studies based on emission surveys of new power plant facilities suggest that organic compounds make the most substantial contributions to CPM (ref. ^7^). One study has revised the emission inventory of Europe by accounting for CPM from residential wood combustion (RWC), and the revision led to two to threefold higher emissions of OA from RWC than previously reported and increased total European PM_2.5_ emissions by ~20% (ref. ^33^). Moreover, in a recent Japanese study, modifying the emission inventory to include CPM from stationary combustion sources resulted in increased emission rates of OA by a factor of seven. Stationary combustion sources in the industrial and energy sectors became the most significant contributors to OA emissions, while road transport and biomass burning were the dominant OA sources in the previous estimate^32^. Taken together, the inclusion of CPM in emission inventories may drastically change emission predictions of UFPs in many sectors.

## Carbon content of UFPs

Black carbon (BC) is defined as carbon with ideally light-absorbing quality, typically formed during incomplete combustion of carbonaceous matter and occasionally by pyrolysis of carbonaceous matter. Elemental carbon (EC) is defined as a substance comprising only carbon and not bound to other elements. Examples of EC are diamond, graphite, carbon nanotubes, or fullerenes. Although BC is mostly composed of EC, BC’s optical characteristic—strong absorption of light within the visible range—is its defining feature, and EC and BC should not be considered synonymous^34^.

OC includes polycyclic aromatic hydrocarbons (PAHs), carbonyl compounds, n-alkanes, organic acids, and heterocyclic compounds. These PAHs and polychlorinated biphenyls may have teratogenic, carcinogenic, and mutagenic properties^35^. OC in PM is produced by two mechanisms: (1) direct emission from sources, and (2) secondary OC formation from VOCs and gas-to-particle conversion of SVOCs (ref. ^36^). EC and OC are determined by thermal/optical methods, while BC is determined by optical or photoacoustic methods^37^. Soot denotes the ensemble of the particles emitted during incomplete combustion, representing a combination of BC and OC (ref. ^34^).

## The challenge of monitoring and characterizing atmospheric UFPs

The condensation particle counter—a device used for the detection of UFPs—was invented more than a hundred years ago^38^. Subsequent development of the photoelectric nucleus counter enabled continuous recording of UFPs with reasonable temporal resolution. Surprisingly, the detectable size limit of the counter was already comparable to modern sensors, reaching well into the sub-10 nm range^39^. However, due to the small size and negligible mass of UFPs, the importance of these particles on human health and disease has historically been underappreciated. Furthermore, due to the monitoring trends based on larger visible particles, the health effects of airborne aerosols have evolved toward mass-based reference limit values, such as PM_10_ or PM_2.5_. Furthermore, mass concentrations correlate variably with UFP concentrations. There is currently no consensus for a standardized method for the measurement or reporting of ambient UFPs, and there are no clear guidelines on acceptable UFP levels.

Collecting UFPs is possible, but due to the extremely low mass of the filtered sample, there are limitations of analyzing their composition and physicochemical characteristics. Moreover, many of the UFPs emitted from fossil fuel combustion are CPM, which may evaporate as they are diluted in the atmosphere, making the analysis more difficult.

## Major sources of anthropogenic outdoor UFPs

Ambient levels of airborne UFPs are intricate to measure both geographically and chronologically because concentrations decline steeply with distance from the sources^24^. Moreover, as previously noted, UFPs grow in size from nucleation to accumulation mode via coagulation and condensation.

In the European Union (EU), the estimate of total UFP emissions in 2008 was 271 kilotons; the sources of UFP emissions were industrial processes (5%), road transport (34%), other transport and machinery (22%), residential and commercial (15%), industrial combustion (12%), power generation (4%), and agricultural sources (8%)^40^. In most urban environments, on-road vehicles are the primary source of UFP emissions. A source apportionment study in London estimated that the total PN concentrations in the city’s ambient air were derived as follows: 65% from vehicle exhaust emissions, 18% from urban background sources, 5% from resuspension, 2% from brake dust, and 10% from other unspecified sources^41^. Occupational exposures are mostly high during high-speed manufacturing, combustion processes, and high-temperature tasks, such as smelting and welding^1^.

In the US, a recent study predicted the regional concentrations of airborne UFP mass in 39 cities across the US during air pollution episodes in the summertime based on the US Environmental Protection Agency’s national emission inventory. Nonresidential natural gas combustion was deemed the chief source of UFPs across the major cities therein. On-road gasoline and diesel vehicles contributed, on average, 14% to regional UFP emissions, which could have been underestimated due to measurement limitations^42^.

However, as mentioned above, these estimates may change significantly in the future if condensable UFPs from industrial stacks are considered contributors, such that proximal industrial sources’ CPM are included as a part of the TPM (ref. ^32^).

## UFPs from diesel exhaust and particle reduction technologies

Motor vehicles, especially those driven by diesel engines, have been specified as a principal source of ambient UFP emission^43^. One recent study on global disease burden estimated that emissions from the transportation sector were linked with 11.4% of total ambient PM_2.5_ and ozone deaths globally in 2015. Among the transportation sources, on-road diesel engines were the major source of emissions^44^. Diesel fuel is used widely not only on traffic roads but in many occupational settings. Approximately 1.4 million workers in the USA and 3 million workers in the EU are occupationally exposed to DE (ref. ^23^).

DE comprises carbonaceous soot particles coated with organic compounds, including alkanes, alkenes, aldehydes, PAHs, and PAH derivatives in addition to inorganic ions, as well as a gas phase consisting of CO, CO_2_, oxides of nitrogen, oxides of sulfur, VOCs, SVOCs, and water vapor^45^.

Solid particles, nonvolatile liquid droplets, and particles formed by condensation are referred to as primary emissions. Several studies have documented that the sulfuric acid derived from the oxidation of sulfur during combustion promotes the nucleation process and increases the number of UFPs (ref. ^46^).

In a recent study, transmission electron microscopy was applied to acquire a reliable picture of the morphology, nature, and chemical composition of nonvolatile UFPs in the exhaust of Euro 6b compliant gasoline and diesel vehicles^47^. The results showed that the UFPs in ash consisted of Ca, S, P, Fe, O, and minor Zn compounds, which may have originated from lubricating oil additives. The ash also had pure Fe oxides, which may have originated from lubricating oil additives, abrasion of engine parts, or combustion of fragments in the combustion chamber. There were more Fe oxides detected in the DE than in the gasoline vehicle exhaust. Some tungsten-bearing particles, which may have originated from the coating layer of the diesel oxidation catalyst (DOC) and the selective catalytic reduction (SCR) catalyst, were recognized in DE. Sub-10 nm particles were mostly attached on ash or enclosed in soot^47^.

There are some effective mass-based strategies for reducing both UFP and BC emissions from diesel and gasoline engines^48^. There are three categories of strategies to control PM emissions: (1) fuel-based strategies, which include reducing sulfur levels; (2) engine-based strategies, which could alter combustion to reduce emissions; and (3) exhaust emission control strategies, which include the use of modern technologies, such as DOCs, diesel particulate filters (DPFs), and SCR catalysts, focusing on reducing emissions after combustion has taken place but before they leave the tailpipe.

Since 1993, European transportation regulations have concentrated on reducing PM mass, CO_2_, and NO_X_ emissions^49^. In addition to these regulations, minimizing sulfur in diesel or gasoline fuel has resulted in reduced PM emissions in all engines^50^. PM reduction is maximized by including both a DOC and DPF in diesel engines. The filtration efficiency for reducing PM emissions has reached >95% and is increasing^51^. The DOC is typically the first device following the engine in the after-treatment system; it is a flow-through catalyst that contains precious metals to initiate the oxidation of HCs, CO, and unburned fuel and oil^52^. The DOC can significantly reduce the amount of semivolatile HC-based condensable UFPs^53^. The catalyst in the DOC, on the other hand, oxidizes NO to form NO_2_, which assists soot oxidation in the DPF, helping the regeneration of the DPF but possibly increasing the NO_2_ emission^52^.

The DPF is a wall-flow filter system that captures any soot and ash particles that the DOC could not oxidize (Fig. 4). The high-efficiency wall-flow DPF is the primary technology used by engine manufacturers to comply with the world’s strictest PM emissions standards. DPFs are currently the best available technology commonly available to reduce the emission of all types of diesel-related PM, BC, and UFPs.

The DPF can filter airborne particles from a gas stream through filtration or physical deposition^54^. Cumulative soot deposition on the DPF walls generates a deep-bed soot cake, which interestingly enhances the filtration efficiency to reduce UPF emissions further. However, despite the positive effects of soot deposition on reducing UFP emissions, heavy deposition can increase the back pressure and decrease the engine efficiency. Regeneration, or oxidizing the deposited particles, is intermittently needed to avoid excessive back pressure on the engine. Through the regeneration process, the soot cake is oxidized into gas, and smaller UFPs are released into the ambient air^49^. Passive regeneration takes place when the temperature within the DPF is between 275 and 360 °C. Active regeneration is activated when sensors detect an excessive build-up of particulates within the DPF. Raw fuel is injected into the exhaust stream and combusted to raise the temperature of the DPF to over 600 °C and remove the soot cake^55^. Active regeneration results in sudden extraordinary emission of UFPs and SVOCs into the ambient air. Experimental results have shown that particle emission during test phases, where filter regeneration takes place can exceed many times the regulatory limit. However, temporary spikes of PN emissions during regeneration are not currently considered in the emission standards^56^. Bimodal particle distributions, which correspond to nucleation and accumulation modes, result during DPF regeneration^57^. The nucleation mode mainly comprises condensed SVOCs originating from the oxidization of accumulated OC (refs. ^58,59^). The accumulation mode during DPF regeneration is associated with carbonaceous soot particles coated with a small fraction of metallic ash and organic compounds^60^. During regeneration, compounds that do not burn or oxidize, such as soot will accumulate in the DPF as ash. Minerals, metals, and other trace elements of the ash most often come from engine wear, breakdown of lubricants, and additives. Some of the metallic ash components are released in exhaust emission with the UFPs (ref. ^47^).

The introduction of DOCs and DPFs in vehicles to lower the tailpipe emissions of HCs and CO had the unanticipated outcome of shifting the particle-size distribution of emitted PM to smaller diameters of 20–30 nm (ref. ^61^). One study documented the impact of a catalyzed DPF on the toxicity of DE (ref. ^62^). In their study, the catalyzed DPF indeed reduced the emitted PM mass and levels of HCs, including carbonyls, as well as VOCs and CO. However, the authors showed that the DPF-filtered exhaust paradoxically resulted in increased injury and inflammation, corresponding with an increase in the levels of NO_2_ and reactive UFPs. The use of the catalyzed DPF led to a 300% increase in the levels of NO_2_ in the exhaust and a 38% increase in the UFP PN count, with a shift in the particle-size mode from 70 nm down to 8 nm. Interestingly, vascular oxidative stress and endothelial dysfunction correlated with the PN count of the UFPs rather than the inhaled particle mass or NO_2_ concentration. Increase in exhaust levels of NO_2_ were also observed in other studies using the catalyzed DPF, which is intended to facilitate the oxidative removal of diesel soot trapped in the filter^63,64^. The substantial reductions in particle mass while increasing the number of smaller sized particles suggested new nucleation mode particles with the modern DPF technology during and after filter regeneration, as has been shown previously^65,66^.

The last element in the after-treatment system is the SCR catalyst, which adds aqueous urea solution into the exhaust system in order to eliminate NO_2_. Despite the increased production of NO_2_ within the DOC or the catalyzed DPF for efficient regeneration, recent advancement in SCR catalysts enables them to capture most of the NO_2_ before leaving the tailpipe^67^. The SCR catalyst not only reduces NO_2_ but may also reduce condensable UFPs. One study described the PM emissions from a stage IIIb nonroad engine that was equipped with a DOC and SCR but lacked a DPF (ref. ^68^). The DOC + SCR decreased the PN concentration by 50% from the engine-out level. The authors noted that the DOC and SCR primarily decreased the formation of condensable UFPs from the volatile organic portion, but not the soot or nonvolatile organic particles that comprised ~34% of the total engine-out PM in the exhaust.

Current European regulation of particle emission limits the amount of PM mass and solid PN. For PN counting, only particles >23 nm are counted. However, studies show that, compared to the number of solid particles >23 nm, the number of solid particles between 10 and 23 nm can be up to 260% higher for gasoline direct injection (GDI) engines, up to 330% higher for traditional gas engines, and up to 60% higher for diesel engines equipped with a DPF (ref. ^69^). Thus, there is a strong argument for lowering the size threshold from 23 nm to 10 nm for solid particles in future regulations. Moreover, despite the abundance of condensable UFPs in nucleation mode, the regulation only considers solid PN, which, as noted previously, is an underestimation of the TPM.

## UFPs from gasoline direct injection engine exhaust

The PM emissions of gasoline vehicles were considerably lower than those of diesel vehicles until the broad introduction of DPFs in the early 2000s. Meanwhile, GDI engines started to become popular in the market due to their improved efficiency over port fuel injection (PFI) engines. Recently, there have been shifts in the gasoline engine market toward turbocharging and GDI engine technology, mainly to improve fuel economy. Such rapid changes have resulted in increased PM mass and UFP numbers. In the conventional PFI engine, fuel is injected into the intake port to form a homogenous air–fuel mixture so that fuel and airflow are injected into the combustion chamber simultaneously and are well mixed. This injection method enables a higher proportion of evaporated fuel and complete oxidization of the fuel. In contrast, with GDI, fuel is sprayed directly into the combustion chamber, which results in an incomplete air–fuel mixture due to the limited time, consequent incomplete combustion, and a higher output of UFPs (ref. ^70^).

Consequently, the PM mass and number emissions of GDI vehicles may also exceed those of diesel vehicles equipped with modern DOCs and DPFs. The rapidly increasing number of GDI vehicles led the EU administrators to make stricter limits for the PM mass levels and a new standard of PN limits for GDI vehicles in the EU, prompting the development of gasoline particulate filters in newer GDI models^71^.

## UFPs from coal-fired power stations

UFPs generated by solid fuel combustion differ in chemical composition from UFPs generated from combustion of gasoline and diesel. In particular, one study showed that UFPs derived from coals were particularly rich in Na, K, Mg, Ca, Ti, Mn, Fe, Co, Ni, Zn, V, Cr, Cu, Sb, As, Se, S, and Cl (ref. ^72^). The authors concluded that UFPs from coal combustion may be chemically more toxic and reactive to the human body because (1) higher concentrations of toxic and volatile compounds were adsorbed in the UFPs than in either the coarse or fine fractions, with enrichments of up to 50-fold; (2) Fe oxides were present in the UFP fraction, which are highly reactive and increase oxidative stress; and (3) unburned carbon, presumably soot, comprised a more substantial fraction in the UFPs than in the coarser fractions. The high carbonaceous content of the UFPs correlated with particle toxicity, possibly due to the increased numbers of oxygenated functional groups on the surface^72^.

The recent changes in anthropogenic clean coal technologies limiting fine particles and NO_X_ emissions have led to a dramatic increase in nucleation mode PN between 5 nm and 15 nm. Thus, coal combustion is now a major contribution to the anthropogenic budget of UFPs in terms of PN, which in turn affects both rural areas and megacities^11,73^. The researchers also found that UFP concentrations have increased continuously, since modern coal-fired power stations were built around the world^74^. The authors revealed that the most persistent UFP sources in the low atmosphere near the surface are modern technology fossil fuel-burning power stations, refineries, and smelters.

## UFPs from outdoor, biomass burning

Massive amounts of PM are generated worldwide annually by biomass burning of billion tons of biomass, such as peat, trees, leaves, and grass. Scrubland and forest fires contribute to 42% of global combustion emissions^75^. Biomass burning is increasingly recognized as a globally significant source of radiatively and chemically active aerosols, containing BC, OC, and inorganic compounds^76^. It has been predicted that these emissions are likely to increase due to climate change, particularly in the boreal forests of North America and Russia^77^, and in the Western USA^78^. This prediction underlines the importance of the proper characterization of biomass burning emissions.

The UFPs found in wildfire smoke are a heterogeneous mixture of chemical species. Wet or green vegetation burns differently than dead and dry vegetation, burning hardwood produces different chemical species than burning softwood, and different stages of combustion (open flame vs. smoldering) produce different chemical profiles^79^. Therefore, the composition of smoke particulates from natural or accidental wildfires burning in a dry season may differ from prescribed burns executed during the wet season^79,80^. Wildfires also have a long smoldering phase, sometimes for months after a fire is considered contained. The smoldering phase of woodburning is associated with long-term output of UFPs and particles in accumulation mode, and can account for a large proportion of the total wildfire air pollutant emissions^80^.

In one study, the size distribution of particles for fuels showed a bimodal distribution, with the smallest mode at ~10 nm, which could be from condensed nucleation of volatiles as the hot gas temperature decreased. The dominant mode of the particle-size distribution was in the range of 29–52 nm for the cycle-averaged distributions, and it was challenging to determine the key parameter that drove particle-size distribution due to the large variability in combustion, humidity, fuel composition, and fuel bed arrangement^81^.

## Major sources of indoor UFPs

Indoor aerosol derives both from the permeation of outdoor particles and from the emissions of indoor sources^82^. There is a wide range of combustion and noncombustion sources that may be active in residential environments, sometimes causing PN concentrations to reach levels well above those encountered in urban environments. Combustion sources include incense, mosquito coil, and candle burning, tobacco cigarette smoke, and cooking activities. Noncombustion sources include domestic appliances driven by brushed electric motors, hot flat irons, spray air fresheners, and heat-not-burn tobacco devices^83–87^. Considerably higher aerosol emissions occur from combustion sources than from noncombustion sources.

Laser printers are one of the major contributors of UFPs in office environments. In one study, researchers measured bimodal number size distributions from laser printer emissions in chamber experiments, with a smaller mode <10 nm and a broader mode extending from ~40 nm up to 100 nm. Moreover, the authors detected UFP emissions with similar size distributions, even when the laser printers were activated without toner or paper. The authors suggested that the high-temperature fuser unit, the device that fixes toner on the paper with heat, was the primary UFP source. They argued that UFP formation proceeds through nucleation and condensation of VOCs or SVOCs released by the fuser, which comprises siloxanes and fluorinated compounds, or emitted from the chassis, which holds flame retardants, lubricants, and plasticizers^88^.

E-cigarette aerosols can be a major source of indoor UFPs, considering today’s popularity of e-cigarette products. Studies have shown that mainstream e-cigarette aerosols contain a significant amount of nicotine and UFPs (refs. ^89,90^). Additionally, VOCs, heavy metals, and carbonyl compounds, possibly generated from the thermal dehydration of glycols and glycerin in the e-liquid, have been detected in mainstream e-cigarette aerosol^91,92^. One study detected two modes at 15 nm and 85 nm when measured 0.8 m away from e-cigarette users^93^. A recent study documented that there were two modes of particle diameters of ~60 nm and 250 nm in busy vape shops with doors closed. The aerosol shifted from a bimodal size distribution to a relatively smaller, unimodal distribution at 40 nm after the door was open^94^. A group of researchers concluded that the inconsistency of particle-size distributions of e-cigarette aerosols might be due to the impacts of e-liquid components, humidity, vaping patterns, and e-cigarette device heating power^95^.

## Effects of UFPs on precipitation and climate change

In one study, researchers performed airborne monitoring near or downwind of coal-fired power stations and over remote regions to study the distribution and transport process of UFPs. Studying the example of smokestacks emitting at heights of 200–300 m or more, UFPs spread over several hundred km depending on weather and climate conditions in the atmosphere^74^. Several studies have calculated the vertical profiles of stack emissions from anthropologic emission sources. The stack height, exit velocity of emission from the stack, and meteorological data would affect the vertical allocation of emissions, which would increase the broad geographic spread of UFPs, as well as the formation of cloud condensation nuclei (CCN). Despite different plume rise calculation models to determine effective emission heights, most calculation results agree that the sector of combustion in energy and transformation industries, which includes coal-fired power stations, had the highest active emission heights, reaching the highest emission layer from 781 m to 1105 m with almost 50% or more of the total emission rising higher than 324 m (ref. ^96^). On the other hand, UFPs emitted from on-road diesel engines may not readily reach high enough to affect cloud formation. Thus, UFPs from modern coal-fired power stations and industrial stacks could have a massive impact on UFP concentrations in the atmosphere over large geographic areas and affect cloud formation.

Model calculations for Western Australia suggest that UFP emissions from a single anthropogenic source may affect regional-scale rainfall, as such UFPs could travel hundreds of km, and grow by coagulation and photochemical reactions^97^. The UFPs grow to sizes of ~40 nm after 2–3 h of travel and become additional CCN. The newly introduced UFPs from smokestacks generate an excessive number of tiny CCN, rather than forming large aerosols. These UFP-induced individual CCN are too small to fall out of the cloud immediately, resulting in decreased steady local precipitation despite the extraordinary increase in the number of CCN (ref. ^74^). However, in some situations, when larger particles are not present high in a warm and humid environment, a high concentration of UFPs may influence the development of thunderstorms and extreme rainfall. When there is a high number of small CCN, they can form many small droplets on which the excess water vapor condenses. A highly humid atmosphere allows the growth of these numerous CCN. The enhanced condensation releases more heat, and the heat makes the updrafts much more powerful. Consequently, more warm air is pulled into the clouds, pulling more droplets aloft and producing more ice and snow pellets, lightning, and rain. Pulling more warm air into updrafts results in the formation of invigorated convection and stronger storms, leading to intense high rainfall in some areas^12^.

Thus, concurrent reduction of cloud droplet size modes by the introduction of excessive UFPs into the atmosphere results in diverse unwanted side effects, such as changes in the distribution and intensity of rainfall on a larger scale, causing either drought or flooding in extreme cases. Such drastic climate change affects the global hydrological cycle and thereby affects global public health both directly and indirectly^11–13,74^.

## Conclusion

UFPs, despite their negligible weight, are present in high numbers and possess a large surface area, allowing a substantial amount of toxic chemicals to be adsorbed. These particles can enter the circulatory system and even enter cellular organelles.

To decrease the total emission of UFPs, more stringent regulations on UFPs should be considered in future emission standards. For on-road vehicles, the contribution of the particle emissions during filter regeneration can be significant, and this should be considered in future regulations. Lowering the size cutoffs for PN counting from 23 nm to 10 nm should be considered and may be possible without large investment costs or significant modifications to existing measurement systems^98^. The current regulation of the solid PN limit should perhaps be extended to the total PN, which includes condensable UFPs from SVOCs. Moreover, to reduce the CPM emission and toxicity of UFPs, all VOCs (not just HCs) should likely be regulated.

However, studies analyzing the composition and health effects of UFPs from various emission sources remain limited relative to those focusing on more traditional size metrics. Despite many reasonable speculations on the toxic role of UFPs on human health, there are not enough data to determine a direct link of their effects on disease morbidity and mortality.

Indeed, there are yet many questions to be answered or refined. Are number-based metrics of UFPs more relevant to disease than those based on mass? Do condensable UFPs affect distal organs after inhalation in a similar way to that of solid UFPs? Are smaller UFPs (e.g., sub-10 nm particles) more toxic than larger UFPs? What are the source-specific long-term effects of UFPs on human health?

There is currently no widely standardized UFP-specific measurement, reporting method, or emission standards. Most official regulations and air quality standards are focused on PM_2.5_, and consequently, major scientific studies are focused on fine particles, which rather inconsistently explain the effect of UFPs. Thus, future studies that focus on the analysis of UFPs may be particularly informative.

There are some issues to consider when considering such studies. First, UFPs are not homogeneously distributed in the atmosphere but instead localized in hotspots of exposure near the emission source due to the sharp PN gradient. Thus, exposure studies and sample acquisition of UFPs should be performed in the vicinity of the specific emission source to represent the real-world effect of exposure. Second, a significant portion of UFPs are CPM, which are not filterable and not measured in many emission sectors but could still contribute as the major source of OA. It is necessary to consider both FPM and CPM to improve the accuracy of emission analysis and forecasting policies for emission sources. Third, more epidemiological studies should focus on UFPs, and widespread UFP emission monitoring networks should be established so that regulatory agencies may develop UFP-specific regulations. Fourth, there should be more consideration given to the effect of UFPs in terms of climate change, especially regarding precipitation, rather than solely focusing on direct health impacts. Recent global climate problems, including extreme drought and flooding, may be in part driven by the recent increase in UFP emissions, and more studies are required to address this issue.
